# Intestinal macrophages and their interaction with the enteric nervous system in health and inflammatory bowel disease

**DOI:** 10.1111/apha.13163

**Published:** 2018-08-12

**Authors:** Elisa Meroni, Nathalie Stakenborg, Maria Francesca Viola, Guy E. Boeckxstaens

**Affiliations:** ^1^ Department of Chronic Diseases, Metabolism and Ageing Translational Research Center for Gastrointestinal Disorders (TARGID) KU Leuven—University of Leuven Leuven Belgium

**Keywords:** cholinergic anti‐inflammatory pathway, enteric nervous system, inflammatory bowel disease, macrophages, vagal nerve stimulation

## Abstract

Over the past decades, there has been an increasing understanding of cellular and molecular mechanisms that mediate modulation of the immune system by the autonomic nervous system. The discovery that vagal nerve stimulation (VNS) attenuates endotoxin‐induced experimental sepsis paved the way for further studies investigating neuro‐immune interaction. In particular, great attention is now given to intestinal macrophages: several studies report the existence of both intrinsic and extrinsic neural mechanisms by which intestinal immune homoeostasis can be regulated in different layers of the intestine, mainly by affecting macrophage activation through neurotransmitter release. Given the important role of inflammation in numerous disease processes, such as inflammatory bowel disease (IBD), cholinergic anti‐inflammatory mechanisms are under intense investigation both from a basic and clinical science perspective in immune‐mediated diseases such as IBD. This review discusses recent insights on the cross‐talk between enteric neurons and the immune system, especially focusing on macrophages, and provides an overview of basic and translational aspects of the cholinergic anti‐inflammatory response as therapeutic alternative to reinstall immune homoeostasis in intestinal chronic inflammation.

## 1 TISSUE RESIDENT MACROPHAGES: KEY PLAYERS IN INTESTINAL HOMOEOSTASIS AND DISEASE

**Figure 1:**
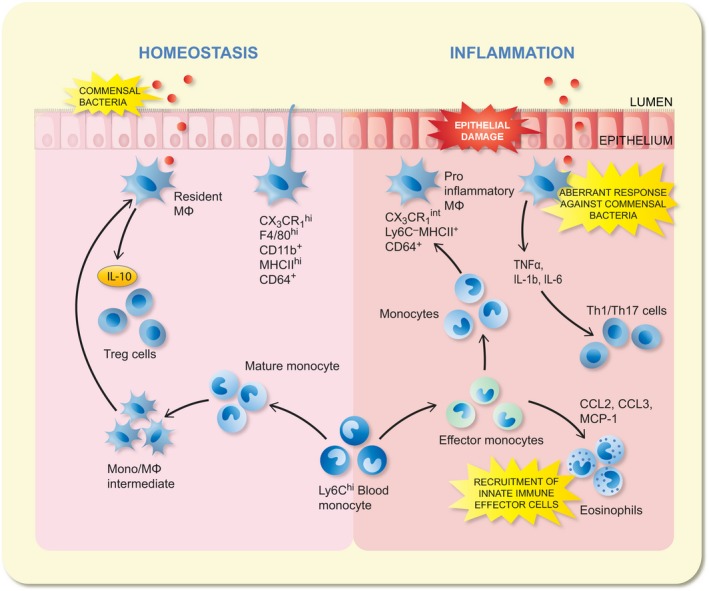
Macrophage differentiation under steady state and disease conditions. Under steady state conditions, Ly6Chi monocytes constitutively enter the intestinal mucosa and differentiate into mature CX3CR1hi F4/80 + Mϕ. These CX3CR1hi Mϕ are found beneath the epithelial barrier where they capture and neutralize invading commensals or pathogens and clear apoptotic cells. Moreover, they are capable of directly sampling the luminal contents; thanks to their extending processes through the epithelial barrier: once the antigen is trapped, it is passed to the CD103^+^ dendtritic cell (DC) which have migratory property. This population is capable to enter in the lymph and reach the mesenteric lymph node (MLN) where can prime T cell. The constitutive production of IL‐10 by the CX3CR1hi Mϕ facilitates secondary expansion of regulatory T cells in the mucosa. When homoeostasis is perturbed by inflammation or infection, Ly6Chi monocytes and CX3CR1int Mϕ accumulate and display pro‐inflammatory characteristics. They produce pro‐inflammatory cytokines (eg TNFα, IL‐1β, IL‐6) which may support the maintenance of other effector cells such as IL‐1/IL‐17‐producing T cells. Moreover, they also orchestrate the recruitment of other innate effector cells such as neutrophils and eosinophils through secretion of inflammatory chemokines (eg CCL2, CCL3)

The gastrointestinal (GI) tract is efficiently organized to protect the host from potential dangerous stimuli and to tolerate commensal microbiota and food antigens. Disruption of normal mucosal immune homoeostasis can potentially lead to uncontrolled chronic inflammation, such as that observed in inflammatory bowel disease (IBD). IBD is characterized by chronic inflammation of the intestinal mucosa with Crohn's disease (CD) and ulcerative colitis (UC) as the two main clinical presentations.^1^ CD is characterized by a transmural inflammation and can affect any part of the intestine. In contrast, the intestinal inflammation in UC is rather continuous and restricted to the mucosal layers of the large intestine. To date, the precise aetiology of IBD remains unknown; however, it is clear that the pathogenesis of both CD and UC is multifactorial, involving immunological, genetic and environmental factors.^2^ For instance, it is known that patients with IBD present an altered immune response to the microbiome.^3^ In recent years it has become clear that the commensal bacteria present in our gut play a fundamental role in intestinal immune development and homoeostasis. Increasing evidence points towards a link between disturbance of the gut microbiota and acute or chronic infections; metagenomic studies showed that intestinal microbiota diversity and stability decrease in IBD patients compared with healthy individuals.^4^ Moreover, an aberrant reaction of immune cells in the GI tract, in particular macrophages (Mφ), towards bacteria and bacterial antigens triggers and drives an exaggerated inflammatory immune reaction.^5^ Intestinal Mφ represent a heterogeneous population of innate immune cells not only playing a crucial role in host defence, but also providing support to the tissue in which they reside.^6^ In the gut, tissue Mφ possess different functions, distinct cell‐dynamics and morphological features depending on their localization.^7^ For example, the lamina propria (Lp) harbours the largest number of Mφ (LpMφ) within the intestine; these cells, characterized by the expression of the receptor CX_3_CR_1,_ are found in close proximity to the intestinal epithelium layer where they surveil the environment, phagocytose potential harmful antigens^8^ and promote epithelial cell renewal by producing several mediators.^9^ As recently demonstrated by Man et al, CX_3_CR_1_^+^ Mφ have the ability to rapidly respond to pathogens by migrating into the intestinal lumen in order to limit the number of bacteria breaching the epithelial barrier.^10^ Furthermore, the expression of receptors for anti‐inflammatory cytokines, such as IL‐10, enable the LpMφ to prevent unnecessary inflammation towards harmless commensal bacteria and instal tolerance to dietary antigens^11^, ^12^ (Figure 1). In contrast to LpMφ, Mφ present in the muscularis externa (MMφ) are located in a dense and intricate network close to the myenteric plexus. Together with the submucosal plexus, the latter represent the intrinsic innervation of the intestine, also referred to as the enteric nervous system (ENS). Finally, although their number is rather low, Mφ are also found within the serosal layer.^7^ These Mφ are larger in size when compared to the resident Mφ and of note, they are not in contact with enteric neurons.^7^ Not only do the different subtypes of Mφ differ with respect to function and location, the heterogeneity of these cells is also reflected in their genetic signature. LpMφ are characterized by a pro‐inflammatory profile which includes high expression levels of *Il1b* and *Il2b* and is shaped by the antigens they encounter arising from the luminal side or the epithelial cells.^7^ In contrast, MMφ have a tolerogenic phenotype characterized by expression of genes such as *Arg1*,* Chi3l3* and *Cd163*.

Given the fact that intestinal Mφ are considered to be the main players in establishing and maintaining gut homoeostasis, loss of tolerance towards commensal bacteria or food antigens is believed to underlie chronic inflammation observed in IBD. Recent preclinical evidence indeed supports an important role for LpMφ in colitis. During inflammatory conditions, ie dextran sulphate sodium (DSS) colitis and T‐cell transfer colitis, there is a marked influx of monocytes and immature Mφ into the mucosal compartment through CC‐chemokine ligand (CCL)2 or MCP‐1 mediated recruitment^9^, ^13^ (Figure 1), an observation which was also confirmed in inflamed mucosal tissue of IBD patients.^14^, ^15^ Interestingly, the differentiation of these incoming monocytes appears to be arrested during inflammation for currently unknown reasons.^14^ As a result, these highly pro‐inflammatory cells are retained in the mucosal compartment, where they produce large amounts of inflammatory mediators, ie IL1, IL6, TNFα, reactive oxygen intermediaries and nitric oxide.^16^, ^17^ Besides causing tissue damage, these mediators also recruit other innate and adaptive immune cells, such as neutrophils, Th1 and Th17 cells.^14^, ^17^, ^18^ Altogether, these findings have renewed the interest in targeting the monocyte‐macrophage lineage for therapeutic purposes in colitis. Especially as monocytes‐macrophages seem to orchestrate the inflammatory process, reducing their activation may prove effective to inhibit the inflammatory cascade during exacerbation of colitis.

## 2 THE CHOLINERGIC ANTI‐INFLAMMATORY PATHWAY

**Figure 2:**
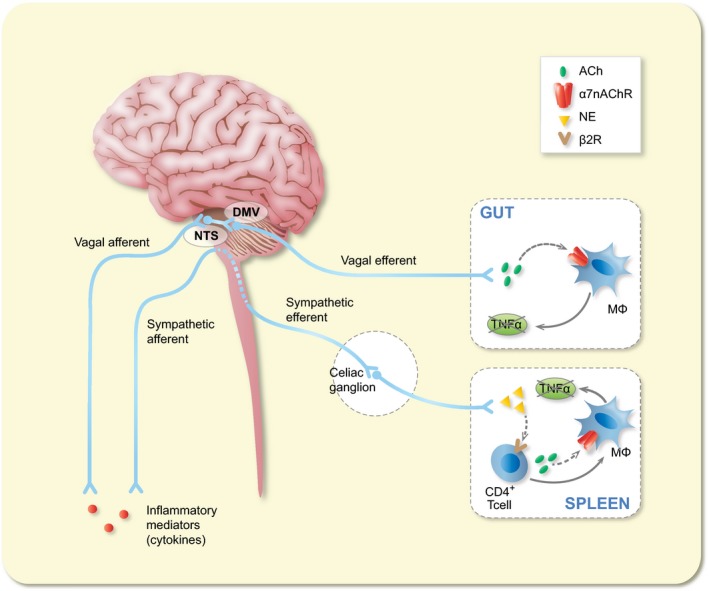
Schematic representation of the cholinergic anti‐inflammatory pathway. Inflammatory mediators, such as cytokines, are released by activated macrophages and other immune cells upon immune challenge. These mediators are detected by the afferent arm of the inflammatory reflex. Efferent vagus nerve cholinergic output to the spleen and gastrointestinal tract regulates immune activation and suppresses pro‐inflammatory cytokine release. Vagal nerve stimulation of the intact vagus nerve stimulates both afferent and efferent fibres. Electrical stimulation of afferent nerve fibres activates neurons in the nucleus of the tractus solitarius, leading to activation of not only efferent vagus nerves but most likely also of an adrenergic pathway resulting in the release of noradrenaline (NA) in the spleen, the major organ source of TNF and other pro‐inflammatory cytokines during endotoxemia and other inflammatory conditions. Here, NE reduces TNF production by splenic macrophages via activation of T cells releasing acetylcholine (ACh) and binding the α7nAChR

In the last decades, the implication of the autonomic nervous system (ANS), and specifically of the parasympathetic innervation, as a key player in immune homoeostasis has increased exponentially. This coincides with the publication of the seminal work by the group of Tracey, which in 2000 showed that the vagus nerve exerts a pivotal anti‐inflammatory input to the immune system, in particular to macrophages, also referred to as the cholinergic anti‐inflammatory pathway (CAIP)^19^, ^20^ (Figure 2). In a model of sepsis, electrical activation of the vagus nerve (VNS) improved survival by dampening splenic TNFα release.^19^ This neuro‐immune interaction was shown to be mediated by neural release of noradrenaline, which stimulates acetylcholine (ACh) release from memory T cells. ACh acts as a mediator, interacting with α7 nicotinic acetylcholine receptors expressed by splenic macrophages.^21^, ^22^ In contrast to the initial hypothesis proposing direct contact between vagal nerve fibres and splenic Mφ,^23^ it is now clear that in the spleen, the vagus nerve rather indirectly modulates the innate immune response by activating adrenergic neurons in the paravertebral ganglia. In line with this hypothesis, only in mice with an intact and innervated spleen, VNS is able to exert its anti‐inflammatory effect.^24^ Given the central role of macrophages in a variety of intestinal diseases (ie post‐operative ileus, gastroparesis and intestinal ischaemia‐reperfusion injury), the therapeutic potential of the CAIP has been a novel and exciting area of GI research.

## 3 CHOLINERGIC MODULATION OF INTESTINAL MACROPHAGES

**Figure 3:**
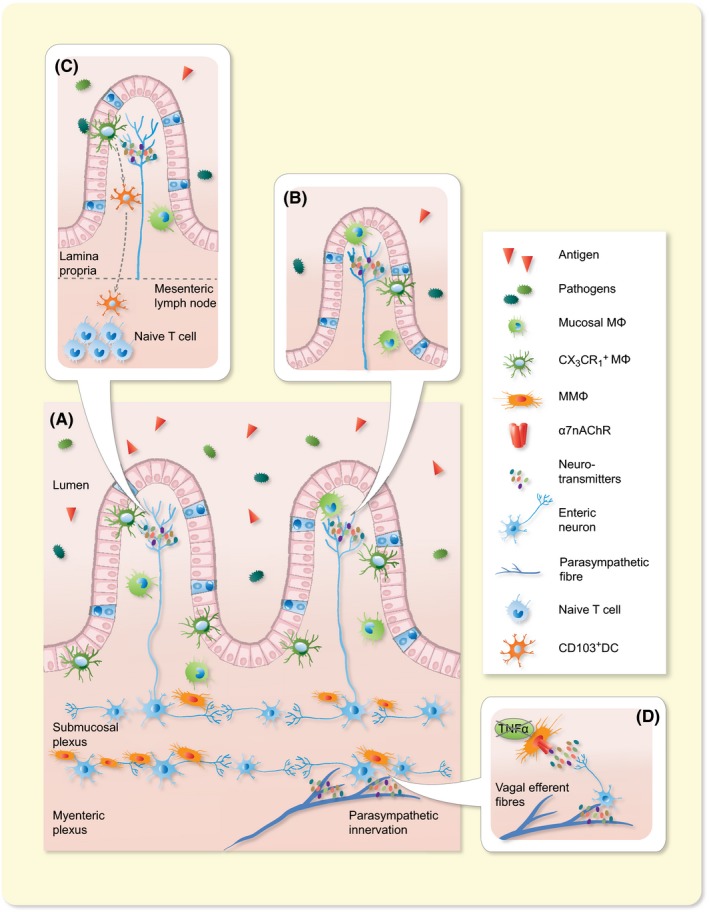
Schematic overview of the cross‐talk between the nervous and the immune system in the GI tract. The gastrointestinal tract is highly innervated by the autonomic nervous system and the enteric nervous system. A, Schematic representation of the intestinal wall with its different layers, showing the distribution of the intrinsic and extrinsic innervation and their relationship with the immune cells. Parasympathetic efferent fibres innervate the intestinal wall by contacting the enteric neurons located in the myenteric plexus region. B, In the mucosal villi immune cells, such as MMϕ and CX3CR1^+^ Mϕ, are instructed by neurotransmitters, like Ach, released by neuronal fibres. C, When the epithelial barrier is crossed by pathogens, CX3CR1^+^ Mϕ are able to migrate into the intestinal lumen in order to rapidly fight the infection and limit the number of bacteria entering in contact with the Lp. Once the antigen is trapped, it is passed to the CD103^+^ dendtritic cell (DC) which have migratory property. This population is capable to enter in the lymph and reach the mesenteric lymph node (MLN) where can prime T cell. D, In the myenteric plexus, close proximity between enteric neurons and resident Mϕ allow these cells to intercommunicate by secretion of neurotransmitters which influence the immune cells’ phenotype, mainly inhibiting TNFα production

The intestinal tract is densely innervated by the vagus nerve. Using anterograde tracers injected into the dorsal motor nucleus of the vagus (DMV), efferent vagal nerve terminals were shown to directly synapse with postganglionic neurons located in the ENS, rather than interacting with neurons in the prevertebral ganglia.^25^, ^26^ The ENS is a complex neuronal network that, in humans, comprises 200‐600 million neurons organized into ganglia.^27^ Hence, the also called “little brain of the gut” controls many GI functions, including motility, secretion, blood flow, mucosal growth and the local immune system. The close contact between nerve fibres and immune cells, especially MMφ, in the gut wall makes an intense reciprocal cross‐talk mediated by a complex set of neurotransmitters, cytokines and hormones, possible^28^, ^29^ (Figure 3). Inflammatory mediators released locally during inflammation have the ability to activate sensory nerves and send signals to the nervous system. In turn, efferent nerves convey signals from the nervous system to the periphery where the release of neural mediators affects the immune response and eventually inflammation.^30^ Consequently, the nervous system is able to rapidly sense and regulate inflammation in peripheral tissues and to restore immune homoeostasis by releasing mediators, which act locally on immune cells. For example, intestinal inflammation, triggered by *Campylobacter jejuni* infection^31^ or intestinal manipulation,^32^ activates neurons of the nucleus of the tractus solitarius (NTS). Goehler et al demonstrated that oral administration of *C. jejuni* induces the expression of c‐Fos, an indirect marker of neuronal activity, in vagal sensory neurons and in the vagal primary relay nucleus in the brainstem. Similarly, the same c‐Fos induction was observed in the NTS in response to intestinal inflammation caused by surgical manipulation of the gut.^32^, ^33^ Interestingly, motor neurons of the dorsal nucleus of the vagus nerve, directly connected to the inflamed area, were also activated, which is compatible with the existence of a hard‐wired inflammatory reflex.^32^ These data indicate that the concept of the “inflammatory reflex”^23^ (ie vagal sensory fibres detecting inflammation in the periphery and sending this information to the brain for integration, leading to the generation of a vagal anti‐inflammatory response) also applies to the intestine. With respect to the latter, cholinergic modulation of intestinal inflammation has indeed been clearly demonstrated in a model of post‐operative ileus.^32^ Here, the muscularis externa becomes inflamed in response to manipulation of the intestine during surgery, a process that is triggered by activation of MMφ and leads to impaired neuro‐muscular performance. The latter explains the subsequent inhibition of intestinal transit in the first days following abdominal surgery, referred to as post‐operative ileus. Of note, VNS dampens MMφ activation, prevents inflammation of the *muscularis externa*, and improves the recovery of GI transit in a murine model of post‐operative ileus.^28^, ^34^ This effect is mediated by activation of cholinergic enteric neurons of the myenteric plexus in close contact with MMφ. Whether VNS results in a similar anti‐inflammatory effect in the Lp remains, however, less well studied. Similar to MMφ, however, cholinergic nerve fibres have also been shown in close proximity of intestinal monocytes‐Mφ at the level of the submucosal plexus and the lamina propria.^28^, ^32^, ^35^ Of interest, efferent vagal terminals do not synapse with intestinal macrophages but with enteric neurons,^36^ pointing towards enteric neurons as possible modulators of intestinal macrophages. Taken together, these observations clearly indicate that the ability of the nervous and the immune system to reciprocally interact offers new opportunities to modulate and treat intestinal inflammation in immune‐mediated disorders of the gut.

## 4 EVIDENCE FOR CHOLINERGIC MODULATION IN INFLAMMATORY BOWEL DISEASE

### 4.1 Patients with inflammatory bowel disease

Autonomic dysfunction with a shift towards increased sympathetic tone and decreased vagal tone has been correlated with a higher risk for the development of chronic inflammatory disorders. Indeed, patients suffering from IBD often present impaired parasympathetic function (ie reduced vagal tone), leading to sympathetic dominance,^37^ potentially contributing to a pro‐inflammatory milieu. In support to this hypothesis, Straub et al showed that the sympathetic tone (ie serum levels of neuropeptide Y) and hypothalamus‐pituitary‐adrenal (HPA) axis (ie serum levels of cortisol) were positively correlated in healthy individuals, but this correlation was abrogated in patients suffering from IBD, who instead show high sympathetic tone and low activity of HPA axis.^38^ Even though the concomitant use of oral steroids may have affected these results, the authors suggested that uncoupling of the sympathetic nervous system and the HPA axis occurs in IBD patients. In addition, the chronically increased levels of systemic and colonic inflammation in active IBD could blunt the release of cortisol in IBD patients, compromising an individual's ability to counter‐regulate mucosal inflammation.^39^ With regard to the impaired vagal activity and the known anti‐inflammatory effects of the vagus nerve, restoring the vago‐sympathetic balance may therefore be pivotal to reduce the recurrence of IBD.

The activity level of the vago‐sympathetic balance and HPA axis can be assessed by peripheral measurements such as the heart rate variability (HRV) and serum cortisol levels respectively. The autonomic functioning can be studied in humans using HRV, a non‐invasive and quantitative electrocardiography‐based technique. Several studies have shown its predictive value in the prognosis of certain chronic inflammatory disorders including rheumatoid arthritis and IBD.^40^, ^41^ For example, Pelissier et al reported that low vagal tone in CD patients was correlated with high levels of serum TNFα and salivary cortisol levels compared to patients with high vagal tone.^42^ This supports the hypothesis that low vagal tone, normally providing inhibitory tonus on cytokine production, contributes to unregulated release of inflammatory mediators.^43^ Notably, the equilibrium of the ANS is differentially regulated in IBD patients, because of emotional adjustment and coping mechanisms. Positive coping is namely correlated with low vagal tone in CD patients, but with high tone in UC patients.^44^ Therefore, IBD patients should be separated according to their disease type, and should also be screened for certain psychological factors when using HRV as a diagnostic marker.^45^ Nevertheless, HRV could serve as an important biomarker to identify patients that may benefit from pharmacological or electrical stimulation of the CAIP. In this context, repeated monitoring of the vagal tone would be useful to determine their efficacy.

### 4.2 Preclinical models of inflammatory bowel disease

Animal studies have confirmed that autonomic imbalance contributes to the inflammatory exacerbation of experimental colitis (Table S1). Of interest, we recently demonstrated that vagotomy (VGX) prevented the development of oral tolerance, the cornerstone of immune homoeostasis. This was associated with reduced induction and expansion of T regulatory (Treg) cells in the mesenteric lymph node (MLNs) and in the intestinal Lp. Further support of an anti‐inflammatory vagal input to the Lp is shown by the observation that mice exposed to DSS after vagotomy (VGX) present an increased susceptibility to develop colitis.^46^, ^47^ During acute DSS‐induced colitis VGX‐mice showed a more pronounced body weight loss and higher stool consistency score compared to non‐VGX‐mice. Similarly, Ghia et al showed that VGX increased the disease activity index in DSS and DNBS colitis models. In addition, myeloperoxidase activity and colonic levels of pro‐inflammatory cytokines were also increased when colitis was induced 9 days after VGX. Of interest, however, at later time points after VGX, the inflammatory response to DSS normalized again, suggesting that the ENS adapts to the decreased vagal input and restores immune homoeostasis. Alternatively, other counter inflammatory mechanisms may come into play if vagal integrity is compromised.^48^

Moreover, adoptive transfer of Mφ from vagotomized mice into Mφ colony‐stimulating factor (M‐CSF) 1 deficient mice exacerbated the severity of colitis, suggesting a central role of Mφ in the vagal anti‐inflammatory effect.^49^ Of note, vagotomized M‐CSF^op/op^ mice did not develop a more severe colitis, highlighting a crucial role for Mφ in the CAIP.^46^ Recently, the pro‐inflammatory effect of vagotomy was also related with a decreased number of colonic and splenic regulatory T (Treg) cells,^50^, ^51^ suggesting that Mφ may negatively affect the development of Treg cells during colitis.

## 5 PHARMACOLOGICAL AND ELECTRICAL STIMULATION OF THE VAGUS NERVE AS TREATMENT OF INFLAMMATORY BOWEL DISEASE

### 5.1 Preclinical evidence

The concept of central activation of the CAIP, either pharmacologically or using VNS, has become very appealing and might be a promising new approach in the treatment of IBD. Up to date, several preclinical studies have provided plenty of evidence supporting a beneficial effect of activation of the CAIP in a variety of disorders. It is indeed of great interest to translate this knowledge into improved clinical management of immune‐mediated inflammatory disease.

The role of ACh in the CAIP is undoubtedly proven: in the spleen, neural release of noradrenaline stimulates ACh release from memory T cells. ACh then, through binding of the α7nAChR expressed by Mϕ, inhibits the release of TNFα from splenic Mϕ thereby dampening inflammation. Therefore, increasing Ach release or prolonging its half‐life may mimic the anti‐inflammatory effect of VNS. In line with this background, several studies have investigated the effect of acetylcholinesterase (AChE) in preclinical models of colitis. AChE is involved in the termination of impulse transmission by rapid hydrolysis of ACh in numerous cholinergic pathways both in the central and peripheral nervous systems. Inactivation of this enzyme leads ACh accumulation and enhanced stimulation of nicotinic and muscarinic receptors. For example, galantamine (GAL) is a reversible, competitive AChE inhibitor, which crosses the blood‐brain barrier, increases brain cholinergic network activity^52^ and is widely used in the treatment of Alzheimer's disease. It has been shown that GAL activates efferent vagus nerve activity^53^ and its anti‐inflammatory property has been associated with brain mAChR‐mediated activation of the CAIP.^54^ Moreover, administration of GAL^55^ ameliorated mucosal inflammation in both DNBS and DSS‐induced colitis, an effect associated with decreased major histocompatibility complex (MHC) II level and pro‐inflammatory cytokine secretion (ie IL‐1β, IL‐6 and TNFα) by splenic CD11c^+^ cells.^55^

In addition, it was shown that the AChE inhibitors neostigmine and physostigmine were able to significantly attenuate macroscopic damage, influx of myeloperoxidase positive cells and smooth muscle thickness in a rodent DNBS model of colitis.^56^

Central activation of the CAIP was also shown through the use of M1 muscarinic acetylcholine receptor (mAChR) agonist McN‐A‐343.^57^ In a recent study, it was demonstrated that central administration of McN‐A‐343 significantly ameliorates the disease activity index in both DNBS and DSS colitis together with decreased levels of IFN‐γ, IL‐1β, IL‐6 and TNF‐α. This effect was mediated through modulation of the functional interaction between DCs and CD4^+^CD25^−^ T cells via α7nAChR and NF‐kB signalling, an effect that was vagus nerve and splenic nerve dependent. Notably, this anti‐inflammatory effect was abrogated in splenectomized mice, suggesting that a possible vagus‐to‐spleen circuitry regulates intestinal inflammation.^55^, ^57^

Interestingly, and in line with the early reports on VNS in sepsis, cholinergic modulation of MMφ by VNS was previously reported to be mediated by α7 nAChR in the small intestine^28^, ^58^ and β2 nAChR in the stomach.^35^ The involvement of this nicotinic receptor, however, still remains a matter of debate in colitis. Although systemic administration of nicotine, a non‐selective agonist for nAChRs, consistently inhibits colonic inflammation in acute DSS colitis,^46^, ^59^, ^60^ contrasting data were reported on the involvement of the α7nAChR during colitis. Several studies demonstrated that male α7nAChR^−/−^ mice developed a more severe DSS‐induced colitis than their respective wild‐type littermates,^55^, ^61^, ^62^ but female α7nAChR^−/−^ mice had a similar disease activity to their littermate controls. Conversely, treatment with (partial) α7nACh receptor agonists (ie choline, PHA‐543613, GTS‐21) lowered DSS‐induced colon inflammation and improved clinical parameters of colitis,^59^, ^61^ but treatment with other specific α7nACh receptor agonists, ie AR‐R17779 and GSK1345038A, worsened disease activity in DSS‐induced colitis because of a higher colonic inflammation.^63^ In addition, the effect of VNS on mucosal immune homoeostasis make use of an alternative molecular pathway compared to the anti‐inflammatory mechanism in the *muscularis externa* and the spleen.^63^ So clearly, to what extent α7nACh receptor agonists can be used to treat patients with IBD needs further study.

In addition to pharmacological activation of the CAIP, the VN can be stimulated by application of electrical pulses. Data on VNS in models of colitis are limited to rats, mainly because the electrodes for chronic VNS commercially available are too big in size to be applicable for implantation in mice. Meregnani et al provided initial preclinical evidence that chronic VNS has therapeutic potential in TNBS‐induced colitis. Using chronically implanted electrodes, electrical stimulation of the vagus nerve was performed 3 hours per day (1 mA, 5 Hz, pulse width of 500 microseconds; 10 seconds on, 90 seconds off; continuous cycle). This treatment alleviated the disease course of TNBS‐induced colitis, including weight loss, bleeding and diarrhoea, leading to remarkable decreases in the disease activity index (DAI) scores. This effect was mediated by inhibition of NF‐kB and mitogen‐activated protein kinase nuclear translocation.^64^, ^65^ Moreover, after 6 days of VNS treatment a pronounced reduction in colonic damage, including the inhibition of inflammatory infiltration and ulcer healing, was observed in VNS‐treated mice compared to sham stimulated. This effect was associated with a progressive restoration of the colonic architecture together with a marked decrease in TNF‐α and IL‐6 production. Interestingly, we recently demonstrated that a single application of VNS to the cervical vagus nerve in mice significantly improves intestinal inflammation and survival in a mouse model of oxazolone‐induced colitis.^66^ VNS applied at the time of induction of oxazolone colitis reduced IL‐6, CXCL1 and TNFα serum levels, and dampened the colonic expression of IL‐6 and CXCL1 (Meroni et al, PLos One, in press). Similarly, we showed that VNS reduced the expression of *TNF*α and *Il6* in intestinal monocytes and increased the expression of *Arg1* in DDS colitis, contributing to improvement in disease activity score (unpublished results). Taken together, these data support VNS as potential novel approach to treat IBD.

### 5.2 Clinical observations

After Tracey et al described the ability of VNS to improve survival in murine model of sepsis, the past 20 years have seen an exponential increase in interest in the therapeutic potential of VNS in immune‐mediated disorders. VNS has become an established FDA‐approved technique and is currently routinely used to treat drug‐resistant epilepsy and depression.^67^ It is classically performed using a spiral electrode wrapped around the left cervical vagus nerve and connected to a pulse generator (Cyberonics) implanted in the left chest wall via a subcutaneous cable.^68^ The left vagus nerve is preferred to the right vagus nerve for electrical stimulation, since it evokes less cardiac effects than the right vagus nerve^68^; indeed, the left vagus innervates the atrioventricular node, while the right vagus innervates the sinoatrial node.

Currently, only few data are available reporting on chronic VNS in patients suffering from IBD. A first attempt was described by Clarencon et al.^69^ A CD patient, treated with low‐frequency VNS (frequency 10 Hz, pulse width 500‐1000 milliseconds, intensity 0.5‐1.5 mA and stimulation on‐time 30 seconds followed by 5 minutes off‐time), showed significant clinical improvement with a reduced clinical disease activity index, reduced inflammation (ie CRP and calprotectin) and endoscopic remission during a 6 month‐long follow‐up period.^69^ This beneficial effect was correlated with an increased parasympathetic tone (ie HRV). VNS was also shown to be safe and well‐tolerated. Most common adverse events included voice alteration, cough, dyspnoea, nausea and headache, which were easily controlled by reducing stimulation intensity. In an open‐label follow‐up study, the same group performed chronic vagus nerve stimulation for 6 months in seven CD patients with active disease. Low‐frequency VNS induced deep remission in 5/7 patients evidenced by significant clinical, biological and endoscopic improvement compared to baseline. Among these seven patients, two were removed from the study at 3 months follow‐up because of clinical worsening.^41^ Another group is also performing an open‐label study with chronic VNS (10 Hz frequency pulse width 250 milliseconds, current 0.5‐2.0 mA for 60 seconds) in CD patients with active disease (n = 8; NCT02951650). So far, 2/5 CD patients reached clinical remission which was associated with reduced levels of calprotectin and endoscopy scoring after 16 weeks of stimulation.^70^ Even though these studies propose a therapeutic role for VNS in active IBD, the findings should be taken with caution considering the power of the study and the fact that a placebo effect cannot be ruled out with an open‐label approach.

Lately, the development of non‐invasive VNS techniques, ie approaches that do not require surgical implantation of an electrode and neurostimulator, are also becoming of interest. These techniques improve the safety and tolerance of VNS and are easy to use, making it more accessible for clinical use. One of these techniques involves transcutaneous activation of the vagus nerve via the auricular concha innervated by vagal afferents.^71^ Several studies showed that transcutaneous VNS (ta‐VNS) can induce a shift in autonomic function towards increased vagal tone^72^, ^73^ and reduce inflammatory cytokine release in whole blood^74^ in healthy volunteers and tinnitus patients. fMRI evidence further substantiated the fact that transcutaneous VNS can activate “classical” central vagal projections including NTS, locus coeruleus, PVN and amydgdala.^75^ Furthermore, physiological modulation of vagal tone using deep slow breathing (DSB) has been shown to prevent the development of acid‐induced oesophageal hypersensitivity in a validated human model.^76^ In fact, DSB has been proposed as a method of inducing analgesia, possibly through increasing parasympathetic nervous system tone through activation of the baroreceptor reflex. In this regard, the ability of DSB in increasing the vagal tone might be of relevance in reducing immune activation.

Currently, two non‐invasive VNS devices are currently available on the market. NEMOS (Cerbomed, Erlangen, Germany) is an external device that provides transcutaneous auricular VNS using an intra‐auricular electrode which stimulates the auricular branch of the VN. The second one, called GammaCore (electroCore LLc, Basking Ridge, NJ, USA), is a non‐invasive VNS device currently being tested for headache, epilepsy and gastrointestinal disorders. It consists of two stainless steel discs that deliver a programmable number of stimulation cycles through a transcutaneous low voltage electrical signal to the cervical VN (120 seconds, 25 Hz).^77^ Clinical studies evaluating the anti‐inflammatory effect of transcutaneous vagus nerve stimulation in inflammatory intestinal diseases are therefore awaited with great interest.

In humans, selective α7nAChR agonists are mostly being evaluated as potential treatments for schizophrenia and Alzheimer's disease. Only a limited number of clinical studies have determined their anti‐inflammatory potential. Of note, the selective α7nAChR agonist GTS‐21 attenuated cytokine production by LPS‐stimulated whole blood in healthy volunteers, and inhibited cytokine release in patients with severe sepsis more potently than nicotine.^78^, ^79^ Nevertheless, GTS‐21 (150 mg three times daily for 3 days) did not significantly reduce the cytokine response in healthy male individuals (n = 14) subjected to experimental endotoxemia (iv administration of 2 ng/kg LPS from *Escherichia coli* O:113) in a double‐blind placebo‐controlled trial.^79^

## 6 CONCLUSIONS

The understanding of the pathogenesis of both CD and UC has considerably increased over the last years. To date, several studies have clarified the involvement of an aberrant immune response in these patients, where macrophages appear to be one of the key players in perpetuating chronic inflammation. Macrophages are specialized phagocytes with crucial roles in the maintenance of intestinal homoeostasis and motility. In the gut, Mφ are divided into different subpopulations depending on their location within the intestine layers and are tightly communicating with the microenvironment. Moreover, in the last decade, the ability of the intrinsic nervous system to modulate the immune response and the possibility to regulate it through VNS technique have obtained great interest.

Current studies are focusing on developing non‐invasive VNS techniques to provide a safer and more durable therapeutics for IBD. Although preliminary data suggest that transcutaneous VNS to treat IBD could prove beneficial, further studies are warranted to better characterize VNS parameters needed to treat chronic inflammation.

## CONFLICTS OF INTEREST

None.
