# Supplementary material for: Intestinal macrophages and their interaction with the enteric nervous system in health and inflammatory bowel disease
Source: Acta Physiol (Oxf). 2018 Aug 12;225(3):e13163. doi: 10.1111/apha.13163 (PMC6519157; doi:10.1111/apha.13163)
Supplement: Supplementary file 1 [file APHA-225-e13163-s001.docx]

| Disease | Model/ treatment | Results | References |
| --- | --- | --- | --- |
| Colitis | Vagotomy and  α7nAChr^-/-^ mice * | Increased colitis severity | Ghia *et al.* 2009; AlSharari *et al.* 2017 |
|  | Vagotomy and α7nAChr antagonist * | Increased colitis severity | Ghia *et al. 2006;* O’Mahony *et al.* 2009; Ji *et al.* 2014 |
|  | Adoptive transfer of Mϕ from vagotomized mice | Increased colitis severity | Ghia *et al.* 2011 |
|  | CD169^+^ MF depletion and neutralizing anti-CCL8 administration | Decreased colitis severity | Asano *et al.* 2015 |
|  | VNS | Decreased colitis severity | Meregnani *et al.* 2011; Sun *et al.* 2013; Meroni *et al.* 2016 |
|  | α7nAChR agonist * | Decreased colitis severity | Bianchi 2007; Ghia *et al.* 2007, 2009, 2011 |
|  | M1mAChR agonist | Decreased colitis severity | Munyaka *et al.* 2014 |
|  | AChE ihibitors | Decreased colitis severity | Miceli & Jacobson 2003; Ji *et al.* 2014 |
|  | Nicotine | Decreased colitis severity | Hayashi *et al.* 2014; Qin *et al.* 2017 |

**Table 1. Overview of studies investigating the role of the CAIP in experimental models of colitis.**

* Contrasting data were reported on the involvement of the α7nAChR during colitis.
